# A Multicenter Retrospective Chart Review of Clinical Outcomes Among Patients With *KRAS* G12C Mutant Non–Small Cell Lung Cancer

**DOI:** 10.1016/j.cllc.2023.01.009

**Published:** 2023-02-08

**Authors:** Wade T. Iams, Meridith L. Balbach, Sharon Phillips, Adrian Sacher, Christine Bestvina, Vamsidhar Velcheti, Xiao Wang, Melina E. Marmarelis, Nan Sethakorn, Ticiana Leal, Paul E. Sackstein, Chul Kim, MD Andrew Robinson, Kathan Mehta, Robert Hsu, Jorge Nieva, Tejas Patil, D. Ross Camidge

**Affiliations:** 1 Vanderbilt University Medical Center, Nashville, TN; 2 Princess Margaret Cancer Centre, Toronto, Ontario, Canada; 3 University of Chicago, Chicago, IL; 4 New York University, NY, NY; 5 University of Pennsylvania, Philadelphia, PA; 6 University of Wisconsin, Madison, WI; 7 Emory University, Atlanta, GA; 8 Georgetown University, Washington DC, USA; 9 Kingston Health Sciences Centre, Kingston, Ontario, Canada; 10 University of Kansas, Kansas City, KS; 11 University of Southern California, Los Angeles, CA; 12 University of Colorado, Aurora, CO

**Keywords:** KRAS G12C mutation, Non-small cell lung cancer

## Abstract

In this large (n = 396), multicenter (n = 13 sites) retrospective chart review of patients with *KRAS* G12C mutant NSCLC we observed a relatively short median rwPFS of 4.6 months among patients with *KRAS* G12C mutant NSCLC treated with docetaxel with or without ramucirumab in the second-line setting, which aligns with the recently reported CodeBreak 200 dataset.

**Background:**

On May 28, 2021, the United States Food and Drug Administration (FDA) granted accelerated approval to sotorasib for second-line or later treatment of patients with locally advanced or metastatic *KRAS* G12C mutant non–small cell lung cancer (NSCLC). This was the first FDA-approved targeted therapy for this patient population. Due to a paucity of real world data describing clinical outcomes in patients with locally advanced or metastatic *KRAS* G12C mutated NSCLC in the second-line or later, we sought to compile a large, academic medical center-based historical dataset to clarify clinical outcomes in this patient population.

**Materials and Methods:**

The clinical outcomes of 396 patients with stage IV (n = 268, 68%) or recurrent, metastatic (n = 128, 32%) *KRAS* G12C mutant NSCLC were evaluated in this multicenter retrospective chart review conducted through the Academic Thoracic Oncology Medical Investigator’s Consortium (ATOMIC). Patients treated at 13 sites in the United States and Canada and diagnosed between 2006 and 2020 (30% 2006–2015, 70% 2016–2020) were included. Primary outcomes included real-world PFS (rwPFS) and overall survival (OS) from time of stage IV or metastatic diagnosis, with particular interest in patients treated with second-line docetaxel-containing regimens, as well as clinical outcomes in the known presence or absence of *STK11* or *KEAP1* comutations.

**Results:**

Among all patients with stage IV or recurrent, metastatic *KRAS* G12C mutant NSCLC (n = 201 with *KRAS* G12C confirmed prior to first line systemic therapy), the median first-line rwPFS was 9.3 months (95% CI, 7.3–11.8 months) and median OS was 16.8 months (95% CI, 12.7–22.3 months). In this historical dataset, first line systemic therapy among these 201 patients included platinum doublet alone (44%), PD-(L)1 inhibitor monotherapy (30%), platinum doublet chemotherapy plus PD-(L)1 inhibitor (18%), and other regimens (8%). Among patients with documented second-line systemic therapy (n = 123), the second-line median rwPFS was 8.3 months (95% CI, 6.1–11.9 months), with median rwPFS 4.6 months (95% CI, 1.4-NA) among 10 docetaxel-treated patients (9 received docetaxel and 1 received docetaxel plus ramucirumab). Within the total study population, 49 patients (12%) had a co-occurring *STK11* mutation and 3 (1%) had a co-occurring *KEAP1* mutation. Among the 49 patients with a co-occurring *KRAS* G12C and *STK11* mutation, median rwPFS on first-line systemic therapy (n = 23) was 6.0 months (95% CI, 4.7-NA), and median OS was 14.0 months (95% CI, 10.8–35.3 months).

**Conclusion:**

In this large, multicenter retrospective chart review of patients with *KRAS* G12C mutant NSCLC we observed a relatively short median rwPFS of 4.6 months among 10 patients with *KRAS* G12C mutant NSCLC treated with docetaxel with or without ramucirumab in the second-line setting, which aligns with the recently reported CodeBreak 200 dataset.

## Background

Oncogenic mutations in *KRAS* occur in approximately 26% and 11% of patients with lung adenocarcinoma in Western and Asian populations, respectively.^1–3^ Until recently, there has been no licensed therapeutic targeting *KRAS* in patients with non–small cell lung cancer (NSCLC). That changed on May 28, 2021, when the United States Food and Drug Administration (FDA) granted accelerated approval to sotorasib for second-line or later treatment of patients with locally advanced or metastatic *KRAS* G12C mutant NSCLC.

This was the first FDA-approved targeted therapy for patients with *KRAS* mutant NSCLC, and it was based on a single-arm study demonstrating a promising objective response rate (37.1%), with a median duration of response of 11.1 months and median progression-free survival (PFS) of 6.8 months^4^ among patients predominantly treated in the third line or later.

There is a paucity of real-world data describing clinical outcomes in patients with locally advanced or metastatic *KRAS* G12C mutated NSCLC in the second-line or later, as most prior studies featured outcomes in the first line setting, evaluated specific patient subgroups, or evaluated the prognostic vs. predictive value of *KRAS* G12C compared to non-G12C genotypes.^5–12^ We sought to compile a large, academic medical center-based historical dataset to clarify clinical outcomes in the second line or later among patients with *KRAS* G12C mutant NSCLC.

## Materials and Methods

### Study Population and Data Collection

The clinical characteristics and outcomes (including demographics, performance status, tumor biomarker testing, treatment types and duration, radiographic results, and follow-up including death) of 396 patients with stage IV (n = 268, 68%) or recurrent, metastatic (n = 128, 32%) *KRAS* G12C mutant NSCLC were evaluated in this multi-center retrospective chart review conducted through the Academic Thoracic Oncology Medical Investigator’s Consortium (ATOMIC). Patients treated at 13 sites in the United States and Canada and diagnosed between 2006 and 2020 (30% 2006–2015, 70% 2016–2020) were included. Data abstraction occurred between August 2020 and June 2021.

### Statistical Analysis

Primary outcomes included real-world PFS (rwPFS) with each line of therapy, with particular interest in the rwPFS when treated with second-line docetaxel, as well as clinical outcomes in the presence of *STK11* or *KEAP1* comutations. The rwPFS was defined as the time between initiation of systemic therapy within the specific line of therapy and documented progression event (radiographic and/or clinical) or death, with censoring at last clinical assessment if subsequently lost to follow-up and no progression event or death was noted upon medical record review. Overall survival (OS) was defined as the time from initiation of systemic therapy to death, with censoring at last clinical assessment if subsequently lost to follow-up and no progression event or death was noted upon medical record review. In order to minimize immortal time bias from molecular testing results (thus excluding potential cases of acquired *KRAS* G12C mutations in subsequent lines of therapy), patient inclusion in any line of treatment rwPFS and OS analysis was restricted to those who had *KRAS* G12C documented any time prior to line of treatment initiation or within 21 days after initiation of the respective line of treatment.

## Results

### Patient Demographics and Follow-Up

Among the 396 patients, 110 (28%) had a *KRAS* G12C mutation identified at the time of initial NSCLC diagnosis, and 286 (72%) had a *KRAS* G12C mutation identified later in their disease course. There were no major demographic differences between patients who had their mutation identified at diagnosis compared to later in their disease course. The median duration of follow-up for the full study cohort was 455 days (15 months). The study cohort included 235 (59%) women and 289 (73%) white patients. Among the study cohort, 371 patients (94%) were current or former smokers (median of 30 pack-year history), and the median age at metastatic disease diagnosis was 67 years old. At the time of metastatic diagnosis, 225 (57%) of patients were classified as Eastern Co-operative Oncology Group Performance Status (ECOG PS) 0 to 1. Patient demographic information is listed in Table 1.

### Patient Clinical Characteristics

Among the study cohort, 268 (68%) patients had stage IV disease at diagnosis, with step wise changes corresponding to initial stage among the recurrent, metastatic cohort—57 patients (14%) with stage III disease at diagnosis, 36 patients (8%) with stage II disease initially, and 29 patients (7%) with initially stage I disease. Among patients with tumor PD-L1 score (TPS) recorded (n = 211 [53%]; due to the historical nature of the dataset, PD-L1 testing was not done in 117 patients [30%] and unknown in the remaining 68 patients [17%]), 57 (27%) had *<*1% expression, 49 (23%) had 1% to 49% expression, and 105 (49%) had 50% or greater expression. Among the full study cohort, 37 (9%) developed brain metastases at any time (OS for brain metastasis cohort included in Supplemental Figure 1) and 28 (7%) developed liver metastases at any time. Detailed clinical characteristics for the full patient cohort are listed in Table 2.

### Overall Study Cohort Outcomes

**Figure 1:**
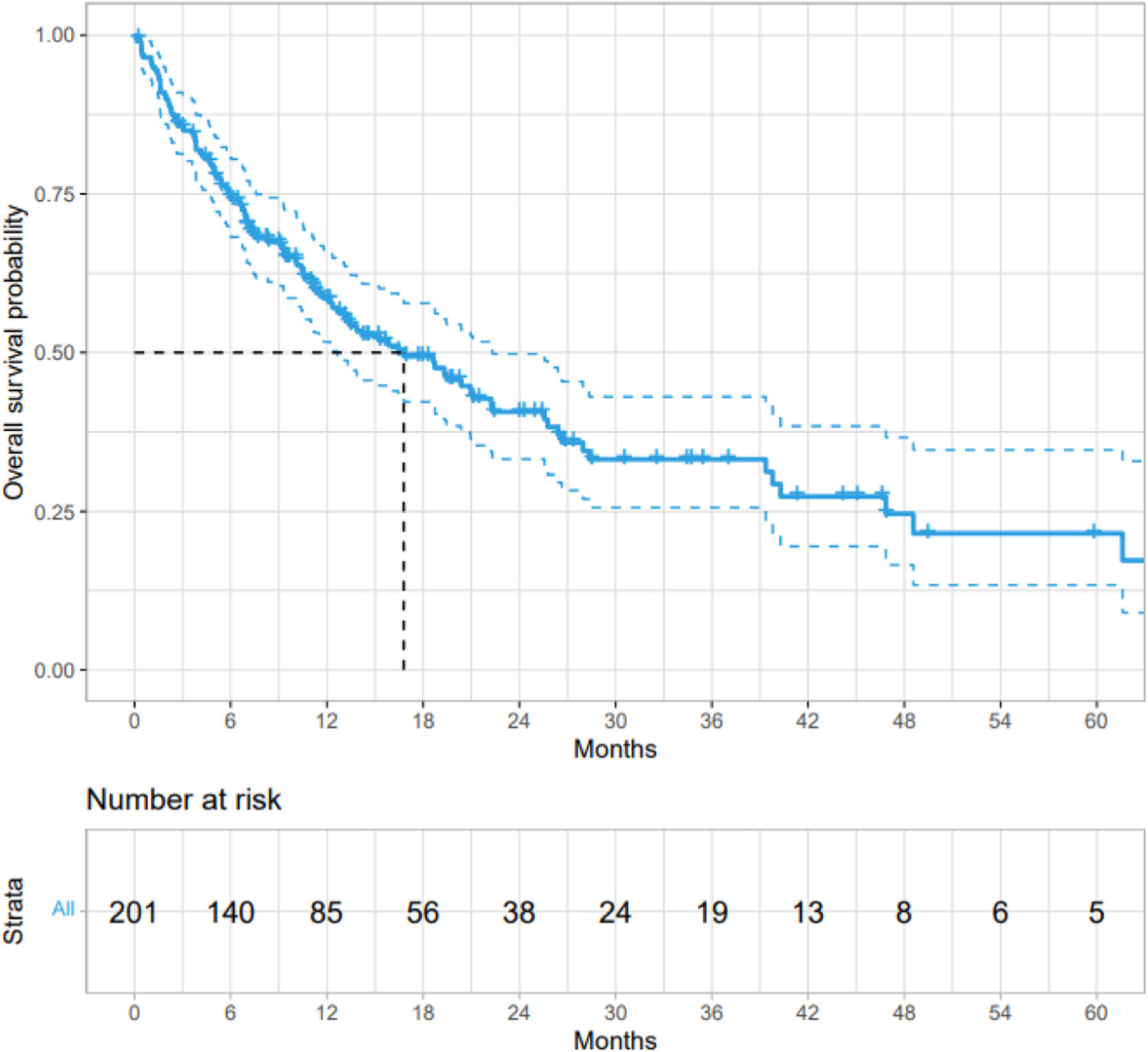
Overall survival on first-line systemic therapy for stage IV or recurrent, metastatic *KRAS* G12C mutant non–small cell lung cancer (n = 201).

Among all patients with stage IV or recurrent, metastatic *KRAS* G12C mutant NSCLC with first line systemic therapy recorded after *KRAS* G12C mutation identification (n = 201), the median first-line rwPFS on systemic therapy for stage IV disease was 9.3 months (95% CI, 7.3–11.8 months) and median OS was 16.8 months (95% CI, 12.7–22.3 months; Figure 1). Treatment data was captured for these 201 patients—in the first-line setting, 89 patients (44%) received platinum-based chemotherapy without a PD-(L)1 inhibitor, 60 patients (30%) were treated with PD-(L)1 inhibitor monotherapy, 36 patients (18%) were treated with platinum based chemotherapy plus a PD-(L)1 inhibitor, and 16 patients (8%) were treated with other regimens. When comparing patients with recurrent, unresectable disease (n = 44) vs. stage IV disease at diagnosis (n = 157) with data on first line systemic therapy, both median first-line OS and rwPFS were similar in patients with recurrent, unresectable disease (median OS: 14.2 months [95% CI, 10.5–26.4 months] vs. 18.7 months [95% CI, 12.4–28.0 months], respectively, and median rwPFS: 10.6 months [95% CI, 7.2–16.5 months] vs. 9.3 months [95% CI, 6.9–12.4 months], respectively).

### Second-Line and Later Therapy Outcomes

Among patients with documented second-line systemic therapy (n = 123), the second-line median rwPFS was 8.3 months (95% CI, 6.1–11.9 months), and the median rwPFS among 10 patients treated with docetaxel with or without ramucirumab (9 received docetaxel and 1 received docetaxel plus ramucirumab) in the second-line was 4.6 months (95% CI, 1.4-NA). Treatment data was captured for 123 patients—in the second-line setting, 55 patients (45%) were treated with other regimens, 39 patients (31%) were treated with PD-(L)1 inhibitor monotherapy, 18 patients (15%) received platinum based chemotherapy without a PD-(L)1 inhibitor, and 11 patients (9%) were treated with platinum based chemotherapy plus a PD-(L)1 inhibitor. Among patients treated with PD-(L)1 inhibitor monotherapy in the second-line setting the median rwPFS was 9.9 months (95% CI, 3.4-NA), among patients treated with platinum based chemotherapy without a PD-(L)1 inhibitor the rwPFS was 8.9 months (95% CI, 6.6-NA), among patients treated with platinum based chemotherapy with a PD-(L)1 inhibitor the rwPFS was 8.0 months (95% CI, 6.1-NA), and among patients treated with other regimens the median rwPFS was 8.3 months (95% CI, 5.1–15.2 months). When second-line therapy outcomes were analyzed by recurrent, unresectable disease (n = 43) vs. stage IV disease at diagnosis (n = 80), rwPFS was similar (median rwPFS 7.7 months, 95% C,I 6.1–15.4 months in patients with recurrent, unresectable disease vs. 8.3 months, 95% CI, 5.5–16.5 months in patients with stage IV disease).

### Comutations and Outcomes

Within the total study population, 49 patients (12%) had a co-occurring *STK11* mutation and 3 (1%) had a co-occurring *KEAP1* mutation. Among the 12% of patients with a co-occurring *KRAS* G12C and *STK11* mutation, median rwPFS on first-line systemic therapy (n = 23) was 6.0 months (95% CI, 4.7-NA), and median OS was 14.0 months (95% CI, 10.8–35.3 months) (Supplemental Figure 2).

## Discussion

This large, multicenter retrospective chart review from 13 academic sites in the United States and Canada of patients with *KRAS* G12C mutant NSCLC confirms similar retrospective data in this patient cohort, which has noted a first-line rwPFS of 4 to 10 months and median OS of 10 to 15 months.^5–9^ In the second-line setting where less robust real world data has been reported and prior retrospective reports have been from a single site, community sites (an important contribution to the literature), or international sites, focused on first line outcomes, specific patient subgroups, or the prognostic vs. predictive value of *KRAS* G12C compared to non-G12C genotypes,^5–12^ in this academic consortium dataset we observed a relatively short median rwPFS of 4.6 months among patients with *KRAS* G12C mutant NSCLC treated with docetaxel with or without ramucirumab. Similar to previous reports,^5^ patients with co-occurring *KRAS* G12C and *STK11* mutations had inferior outcomes with first-line immune checkpoint inhibitor (in this historical dataset, approximately half of patients were treated with immune checkpoint inhibitors in the first line).

Limitations of the study include its retrospective nature, potential immortal time bias in the subgroup OS estimate for brain metastasis at any time, lower than expected frequency of brain metastases compared to other retrospective series in patients with *KRAS* G12C mutant NSCLC,^10,13–15^ and that patient numbers among those treated with docetaxel were limited as all patients were treated in academic medical centers and more often enrolled in clinical trials in the relapsed NSCLC setting. Furthermore, standard of care first line therapy changed throughout the treatment interval included in this retrospective series, resulting in just 18% of patients in this cohort receiving first line chemoimmunotherapy, limiting generalizability to the current treatment landscape.

## Conclusion

In conclusion, our real-world data suggest a short rwPFS (less than 5 months) among patients with *KRAS* G12C mutant NSCLC treated with docetaxel containing regimens in the second line, which is congruent with recently reported results from CodeBreak 200 (a randomized trial comparing sotorasib vs. docetaxel in this patient population).^16^

## Supplementary Material

1

## Figures and Tables

**Table 1:** Patient Demographics of Full Cohort of Patients With *KRAS* G12C Mutant Non–Small Cell Lung Cancer

Demographic	Patients (N = 396)
Sex	
Male	161 (40.66%)
Female	235 (59.34%)
Age at metastatic NSCLC diagnosis	
18–64 y	169 (42.68%)
65–74 y	139 (35.10%)
75–84 y	77 (19.44%)
*>* = 85 y	11 (2.78%)
Age at metastatic diagnosis	
Median (IQ range)	67.35 (59.64–74.36)
Range	26.30–93.20
Race	
White	289 (72.98%)
Black	57 (14.39%)
Asian Indian	1 (0.25%)
Chinese	5 (1.26%)
Korean	2 (0.51%)
Other Asian	3 (0.76%)
Other—not listed above	5 (1.26%)
Unknown	34 (8.59%)
Smoking status	
Current smoker	85 (21.46%)
Former smoker	286 (72.22%)
Never smoker	18 (4.55%)
Unknown	7 (1.77%)
ECOG score at metastatic disease	
0	71 (17.93%)
1	154 (38.89%)
2	41 (10.35%)
*>* = 3	130 (32.83%)
Not available	0 (0 ·00%)

**Table 2:** Clinical Characteristics of Full Cohort of Patients With *KRAS* G12C Mutant Non–Small Cell Lung Cancer

Clinical Characteristic	Patients (N = 396)
Year of metastatic NSCLC diagnosis	
*<*2011	15 (3.79%)
2011	18 (4.55%)
2012	9 (2.27%)
2013	6 (1.52%)
2014	16 (4.04%)
2015	33 (8.33%)
2016	50 (12.63%)
2017	56 (14.14%)
2018	68 (17.17%)
2019	83 (20.96%)
2020	42 (10.61%)
Stage at initial NSCLC diagnosis	
Unknown	6 (1.52%)
Stage Ia	29 (7.32%)
Stage IIa	13 (3.28%)
Stage IIb	23 (5.81%)
Stage IIIa	40 (10.10%)
Stage IIIb	15 (3.79%)
Stage IIIc	2 (0.51%)
Stage IV	268 (67.68%)
Histology	
Adenocarcinoma	217 (54.80%)
Squamous cell carcinoma	5 (1.26%)
Adenosquamous carcinoma	4 (1.01%)
Large cell carcinoma	2 (0.51%)
Non–small cell carcinoma, NOS	24 (6.06%)
Other	4 (1.01%)
Not specifically noted	140 (35.35%)
PD-L1 grouping	
*<*1%	57 (27.01%)
1%−49%	49 (23.22%)
≥50%	105 (49.76%)
Data not available	185 (46.72%)
TMB	
*<*10 mutations/megabase	26 (60.47%)
≥10 mutations/megabase	17 (39.53%)
Data not available	353 (89.14%)
CoMutations	
*TP53*	152 (38%)
*STK11*	49 (12%)
*KEAP1*	3 (1%)
*EGFR*	∗n = 1 with L858R, n = 1 with T790M
ALK rearrangement	1 (0.3%)
Brain metastases at diagnosis	
Yes	28 (7.1%)
No	368 (92.9%)
Brain metastases at any time	
Yes	37 (9.34%)
No	359 (90.66%)
Adrenal metastases at any time	
Yes	38 (9.60%)
No	358 (90.40%)
Bone metastases at any time	
Yes	76 (19.19%)
No	320 (80.81%)
Liver metastases at any time	
Yes	28 (7.07%)
No	368 (92.93%)
Lung metastases at any time	
Yes	137 (34.60%)
No	259 (65.40%)
ECOG score at second line of treatment start	
0	14 (14.00%)
1	67 (67.00%)
2	16 (16.00%)
*>* = 3	3 (3.00%)

## Clinical Practice Points

- Patients with KRAS G12C mutant NSCLC experience a real-world progression-free survival (rwPFS) of approximately 9 months in the first-line and 4 to 5 months in the second-line setting when treated with docetaxel containing regimens.
- Patients with KRAS G12C mutant NSCLC with a co-occurring STK11 mutation experienced inferior outcomes when treated with immune checkpoint inhibitors.
