# Supplementary material for: A Multicenter Retrospective Chart Review of Clinical Outcomes Among Patients With KRAS G12C Mutant Non–Small Cell Lung Cancer
Source: Clin Lung Cancer. Author manuscript; Available in PMC 2024 May 1. (PMC10234144; doi:10.1016/j.cllc.2023.01.009)

Supplemental Figure Legends

Supplemental Figure 1. Overall survival curve for patients with *KRAS* G12C with brain metastasis at any time (n=37).

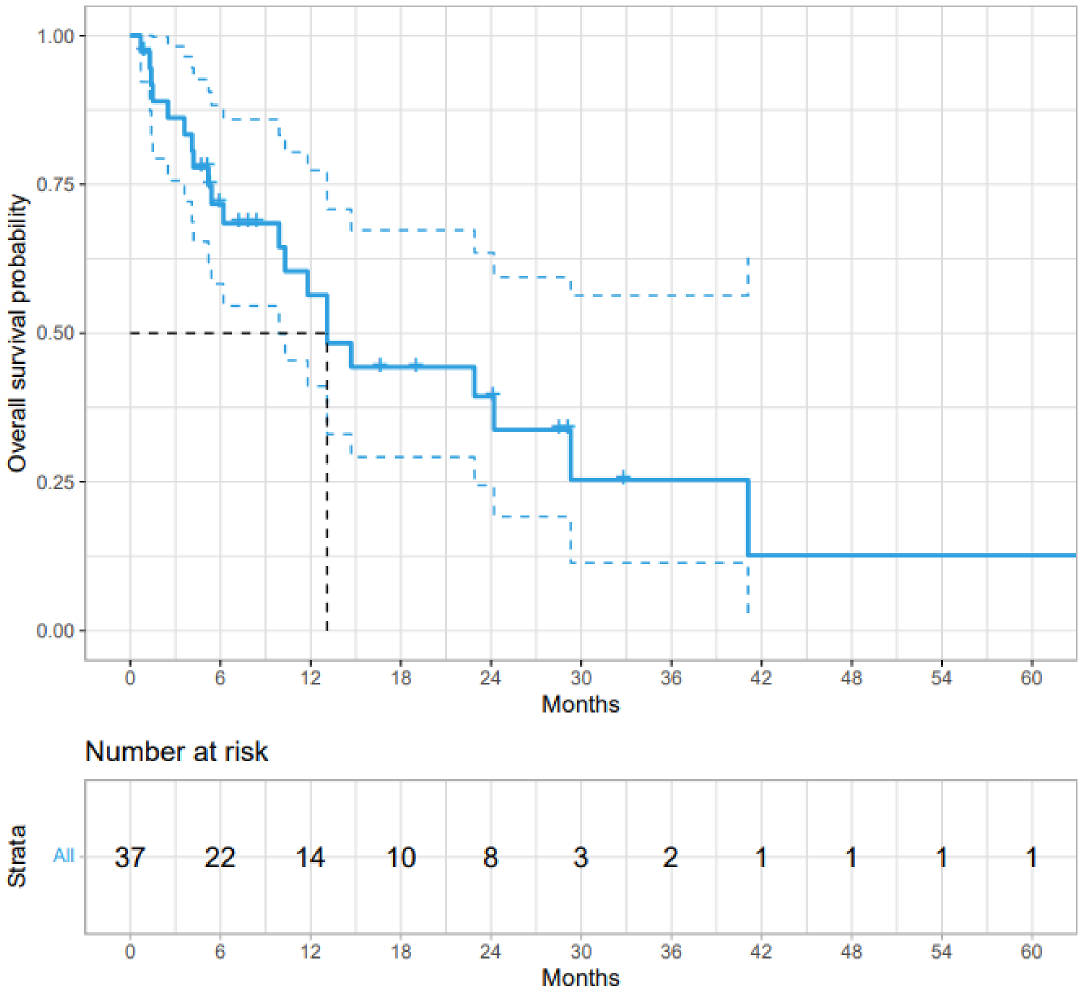

Supplemental Figure 2. Overall survival curve for patients with *KRAS* G12C with co-occurring *STK11* mutation (n=49).

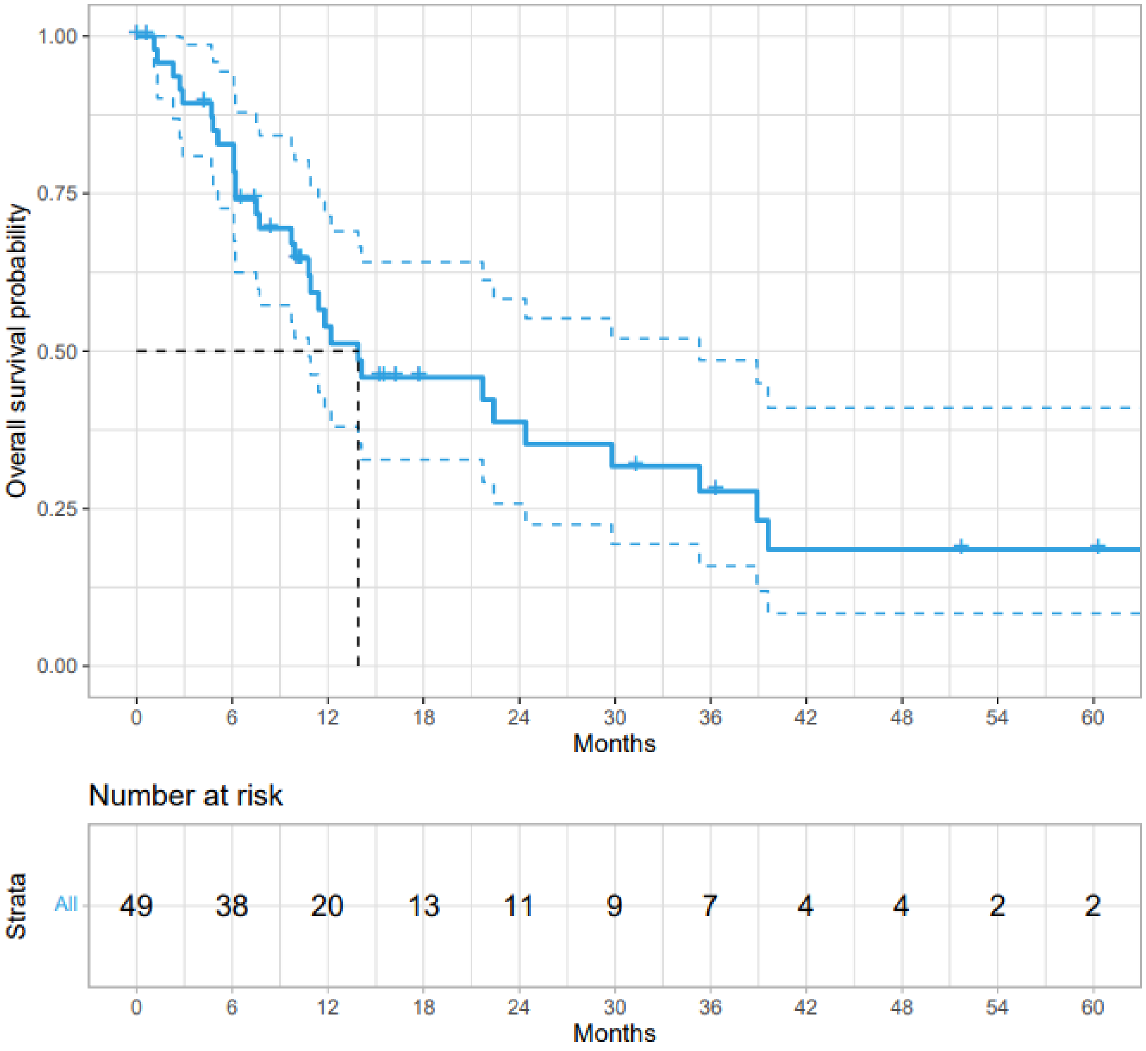

Supplement: 1 [file NIHMS1895210-supplement-1.pdf]
